# Fetal Alcohol Spectrum Disorders: Research Challenges and Opportunities

**Published:** 2011

**Authors:** Kenneth R. Warren, Brenda G. Hewitt, Jennifer D. Thomas

**Keywords:** Fetal alcohol spectrum disorders, fetal alcohol syndrome, fetal alcohol effects, maternal alcohol consumption, prenatal alcohol exposure, pregnancy, alcohol related birth defects, developmental disorders, diagnosis, research, alcohol-related neurodevelopmental disorder

## Abstract

The adverse effects of prenatal alcohol consumption have long been known; however, a formal description and clinical diagnosis of these effects was not introduced until 1973. Since then, the distinction of the wide range of effects that can be induced by prenatal alcohol exposure, and, consequently, the terminology to describe these effects has continued to evolve. Although much progress has been made in understanding the consequences of prenatal alcohol exposure, challenges still remain in properly identifying all affected individuals as well as their individual patterns of alcohol-induced deficits. Also, as the large numbers of women who continue to drink during pregnancy indicate, prevention efforts still require further refinement to enhance their effectiveness. In addition, the mechanisms underlying alcohol-induced damage have not yet been fully elucidated; as knowledge of the mechanisms underlying alcohol-induced deficits continues to grow, the possibility of minimizing potential harm by intervening during prenatal alcohol exposure is enhanced. Finally, researchers are exploring additional ways to improve or fully restore behavioral and cognitive functions disrupted by prenatal alcohol exposure by treating the individuals with fetal alcohol spectrum disorders, thereby reducing the heavy burden for affected individuals and their families.

Although the effects of alcohol consumption on pregnancy outcomes have been observed throughout history, alcohol’s ability to interfere with embryonic and fetal development (i.e., teratogenicity) was not recognized until the latter half of the 20th century. Alcohol now is recognized as the leading preventable cause of birth defects and developmental disorders in the United States (Bailey and Sokol 2008). The severity of birth defects resulting from exposure of the developing embryo or fetus to alcohol is determined by multiple factors, including genetic background, timing and level of alcohol exposure, and nutritional status (Guerri et al. 2009; Warren and Li 2005). The most serious adverse consequence of prenatal alcohol exposure is fetal alcohol syndrome (FAS), which has an estimated prevalence that ranges from 0.5 to 7.0 cases per 1,000 births in the United States (May and Gossage 2001; May et al. 2009). Moreover, many children who have been exposed to large amounts of alcohol prenatally may exhibit alcohol-related brain and behavioral abnormalities but are not diagnosed with FAS because they do not show the abnormal facial features. This range of deficits now is referred to as fetal alcohol spectrum disorders (FASD) and is estimated to occur in 1 percent of births (Sampson et al. 1997), although some have suggested that the rate is much higher (May et al. 2009). The disabilities associated with FASD can persist throughout life and place heavy emotional and financial burdens on individuals, families, and society.

This is the third issue of *Alcohol Research & Health* and its predecessor, *Alcohol Health & Research World*, to address the problems resulting from prenatal alcohol exposure (National Institute on Alcohol Abuse and Alcoholism [NIAAA] 1994, 2001), and it is clear that although significant progress has been made, many challenges remain. For example, researchers have made progress both in understanding the mechanisms of how alcohol damages the fetus, including epigenetic influences, and in the potential prevention of such damage through the development of pharmacotherapeutic interventions. Nevertheless, too many pregnant women continue to drink and too many nonpregnant women in their childbearing years drink in risky patterns that place themselves and their potential progeny at high risk for negative health outcomes, making prevention and intervention strategies a high priority.

This article tracks the changing concepts and refinements in diagnoses that have occurred since the first description of FAS in 1973; next, it highlights some of the research advances made in recent years in diagnosing the effects of fetal alcohol exposure, elucidating the mechanisms through which alcohol exerts its detrimental effects, preventing prenatal alcohol exposure, and developing treatments for affected individuals. These highlights underscore the fact that although knowledge of the consequences of fetal alcohol exposure and their underlying mechanisms has expanded significantly, the field still faces continuing challenges, and the goal of ameliorating these life-long consequences has not yet been accomplished.

## Diagnosing the Effects of Fetal Alcohol Exposure

As can be expected in any scientific or medical field, the terminology used to define and diagnose the adverse effects of prenatal alcohol exposure has evolved since the first formal description of FAS (Jones and Smith 1973; Lemoine et al. 1968). The only diagnosis of an effect of prenatal alcohol exposure that currently is widely accepted remains the full presentation of FAS. Despite some refinements, the essential diagnostic criteria for FAS have changed very little since they were first described in the literature (Centers for Disease Control and Prevention [CDC] 2004; Warren and Foudin 2001). Several significant attempts have been made over the years to categorize the effects of prenatal alcohol exposure that do not meet guidelines for FAS. The criteria for diagnosing FAS and other terms developed by consensus among stakeholders (e.g., researchers, Federal agencies, medical organizations, patient advocacy organizations, etc.) to describe the wide range of effects of prenatal alcohol exposure are summarized in the following paragraphs.

### Terminology Used to Describe Alcohol’s Effects

The evolution in the understanding of alcohols effects on the fetus has resulted in a variety of (sometimes overlapping) terms that have been used to characterize the range of alcohol’s potential effects. These include FAS, partial FAS (pFAS), fetal alcohol effects (FAE), alcohol-related birth defects (ARBD), alcohol-related neurodevelopmental disorder (ARND), and fetal alcohol spectrum disorders (FASD).

#### FAS

*A* diagnosis of full FAS is made if the following three primary defining features are present:

- Documentation of characteristic facial abnormalities (smooth philtrum, thin vermillion border, and short palpebral fissures) (see figure);
- Documentation of prenatal and postnatal growth deficits; and
- Documentation of central nervous system (CNS) abnormalities (i.e., structural, neurological, or behavioral, or a combination thereof).

#### FAE

The term suspected “fetal alcohol effects” (FAE) was introduced in 1978 as a label for the negative outcomes of alcohol exposure during pregnancy that failed to meet all the criteria for FAS (Clarren and Smith 1978). In 1980, this designation was reaffirmed by the Research Society on Alcoholism’s Fetal Alcohol Study Group (FASG) (Rosett 1980). However, two problems developed with the use of this terminology. First, researchers began to use terms such as “suspected FAE” in the literature as a diagnosis rather than as a “bookmark” that merely suggests that the abnormalities seen in the child were compatible with those caused by prenatal alcohol exposure but were not sufficient for a diagnosis of FAS (Aase et al. 1995). Second, the term FAE was too broad and began to be used indiscriminately by clinicians and other entities (e.g., health and other care providers and agencies) eager to obtain needed services for their patients, clients, and children (Aase et al. 1995; Hoyme et al. 2005). The FASG subsequently strongly discouraged the use of the term FAE in scientific publications (Sokol and Clarren 1989), and, eventually, the authors of the article that originally had proposed the term FAE recommended abandoning its use in clinical settings (Aase et al. 1995).

#### ARBD and ARND

At its 1987 meeting, the Research Society on Alcoholism’s FASG attempted to agree on a more precise terminology for describing alcohol’s broad effects on the fetus. Although this attempt was not successful, the group did reaffirm the diagnostic criteria for the FAS and suggested that the term “alcohol-related birth defects” (ARBD) be used to describe “observed anatomic or functional outcome to the impact of alcohol on the offspring” (Sokol and Clarren 1989, p. 598). Subsequently, ARBD came to refer more specifically to physical anomalies associated with prenatal alcohol exposure (Stratton et al. 1996, see below).

The term “alcohol-related neurodevelopmental disorder” (ARND) is used to describe individuals with confirmed prenatal alcohol exposure who exhibit CNS neurodevelopmental abnormalities and/or evidence of complex patterns of behavioral or cognitive abnormalities that cannot be explained by other genetic or environmental factors (Chudley et al. 2005; Hoyme et al. 2005; Stratton, et al. 1996). Because they do not exhibit the physical features associated with prenatal alcohol exposure, individuals with ARND may be more challenging to recognize and differentiate from individuals with other developmental disorders. The elucidation of patterns of neurobehavioral effects of prenatal alcohol exposure that will facilitate identification is an ongoing goal (for more information, see the articles by Coles, pp. 42–50, and by Mattson and Riley, pp. 51–55, in this issue).

The terms ARBD and ARND are used as one of five diagnostic categories recommended by the Institute of Medicine (IOM) in a 1996 report (Stratton et al. 1996). These categories include the following:

- FAS with a history of maternal alcohol exposure;
- FAS without a history of maternal alcohol exposure;
- Partial FAS with a history of maternal alcohol exposure, which includes people with signs and symptoms attributable to significant prenatal alcohol exposure who need medical, social services, and other attention but who would not receive a diagnosis of FAS with confirmed maternal alcohol exposure;
- ARBD, which refers to people with alcohol-related physical anomalies only; and
- ARND, which refers to people who manifest neurodevelopmental, cognitive, or behavioral abnormalities attributable to prenatal alcohol exposure.^1^

In this diagnostic scheme, ARBD, along with partial FAS and ARND, constitute the broad category of alcohol-related effects—that is, clinical conditions that clearly have been linked (through clinical or animal studies) to maternal alcohol ingestion. These categories, along with the specific IOM-recommended diagnostic criteria for FAS and alcohol-related effects, were described fully in the previous issue *of Alcohol Research & Health* focusing on these outcomes (Warren and Foudin 2001) that is available on the NIAAA Web site at http://pubs.niaaa.nih.gov/publications/arh25-3/153-158.htm.

### FASD

In 2003, the National Task Force on Fetal Alcohol Syndrome and Fetal Alcohol Effect (NTF FAS/FAE), a Federal advisory committee with membership from across several Federal health agencies and the public, developed guidelines for the diagnosis of FAS. The product, *Fetal Alcohol Syndrome: Guidelines for Referral and Diagnosis*, provided detailed standard diagnostic criteria for the facial abnormalities, growth deficits, and CNS abnormalities of FAS and was intended to promote consistency in diagnosis for clinicians, scientists, and service providers (Bertrand 2004). The *Guidelines* also included a consensus statement on the definition of FASD that was developed at an April 2004 summit. That statement, provided below, included input from Federal agencies and experts in the field and was sponsored by the National Organization on FAS (Bertrand 2004, p. 4):

“Fetal alcohol spectrum disorders (FASD) is an umbrella term describing the range of effects that can occur in an individual whose mother drank alcohol during pregnancy. These effects may include physical, mental, behavioral, and/or learning disabilities with possible lifelong implications. The term FASD is not intended for use as a clinical diagnosis.”

### Diagnostic Criteria for Describing the Consequences of Prenatal Alcohol Exposure

Several major published sets of diagnostic criteria for FAS and other negative effects of prenatal alcohol exposure currently are being used to identify and describe individuals exposed to alcohol prenatally. The IOM guidelines (Stratton et al. 1996) proposed a set of descriptions for the five disease categories outlined in the guidelines; however, these descriptions were not operationalized for use in clinical settings. A more detailed diagnostic system, commonly referred to as either the 4-Digit Code or the Washington Criteria, was developed by Astley and Clarren (2000). In 2005, Hoyme and colleagues (2005) published a more refined set of diagnostic criteria, derived primarily from those of the IOM, that are intended to be more applicable to clinical pediatric practice (Hoyme et al. 2005). Likewise, the Public Health Agency of Canada’s National Advisory Committee on Fetal Alcohol Spectrum Disorder issued a set of diagnostic guidelines (Chudley et al. 2005). A comparison summary of these diagnostic schemata, including the guidelines generated by the National Task Force on Fetal Alcohol Syndrome and Fetal Alcohol Effect (Bertrand et al. 2004) is shown in the table.

The terminology used in describing and diagnosing the consequences of prenatal alcohol exposure continues to be fluid, as can be expected in any area of medicine, and likely will continue to evolve as new and more refined evidence becomes available.

## Progress and Challenges for FASD Research

### Case Ascertainment

As a result of the refinements in clinical criteria for diagnosing FAS and other effects of prenatal alcohol exposure over the past 35 years, the ascertainment of cases for both clinical and research purposes has improved. Nonetheless, the subtle nature of some of the dysmorphic features associated with prenatal alcohol exposure continues to pose difficulties, particularly for diagnosis in infancy. Failure to recognize alcohol-related dysmorphology and resultant underreporting may interfere with affected individuals receiving appropriate medical and social services. Advances in three-dimensional computer recognition of FAS dysmorphology, using algorithms for feature detection from facial images (Fang et al. 2008) and stereo photogrammetry (Mutsvangwa et al. 2009), are promising tools that eventually may improve diagnosis, particularly among populations who do not have access to a specialist trained in recognizing dysmorphology. (For more information on the three-dimensional analysis of FASD features, see the sidebar by Wetherill and Foroud, pp. 38–41, in this issue.)

Another key challenge facing clinicians is the ability to identify FASD when no or only partial dysmorphic features are present. Although computer-generated imaging may detect subtle dysmorphology, biomarkers that indicate exposure to alcohol during the prenatal period would provide an additional tool to identify individuals with FASD and to better relate alcohol exposure parameters with outcomes. The majority of alcohol ingested by the mother and the fetus eventually is oxidized to carbon dioxide and water, neither of which could serve as a marker for alcohol exposure. Recently, however, there have been advances in methodologies to measure breakdown products (i.e., metabolites) of alcohol that are produced when alcohol is broken down through other pathways (Burd and Hofer 2008; Pragst and Yegles 2008). These metabolites persist in various tissues (e.g., blood, urine, and hair) for multiple days to weeks after alcohol exposure, providing new opportunities for monitoring alcohol exposure in either the newborn or the mother. Other potential biomarkers include those that derive from alcohol-induced alterations in the individual’s metabolic, proteomic, or epigenetic profile. Although continued use and refinement of maternal drinking questionnaires (e.g., the T-ACE or AUDIT-C) will provide valuable information regarding alcohol exposure levels, reliable biomarkers that eliminate the need to depend on self-reporting of alcohol consumption during pregnancy will enhance both diagnosis and understanding of the relationship between alcohol exposure levels and outcome, particularly because self-report data may be inaccurate or unavailable. Furthermore, biomarkers reflecting altered biochemical profiles also may help to describe the extent of the FASD injury itself. (For more information, see Bakhireva and Savage, pp. 56–63, in this issue.)

### Consequences of Prenatal Alcohol Exposure

A better understanding of the physical, neurological, and behavioral patterns of alcohol’s effects also can help improve diagnosis of affected individuals. For example, elucidation and enhanced understanding of specific neurodevelopmental consequences of prenatal alcohol exposure would enhance the clinicians’ ability to distinguish FASD from other developmental disabilities. Comparisons with other developmental disorders are key for generating diagnostic tools that are both sensitive and specific (see Coles, pp. 42–50 and Mattson and Riley, pp. 51–55, in this issue).^2^ Moreover, recognizing the relative strengths and weaknesses in patients’ cognitive/behavioral profiles will help researchers and treatment providers design interventions that target weaknesses while using strategies that engage existing strengths.

Imaging technologies, such as magnetic resonance imaging (MRI), are helping to elucidate alcohol’s neuropathological effects. Because of the lifelong learning and neurobehavioral deficits that characterize FAS and other types of FASD, the CNS unquestionably is the most critical system adversely affected by prenatal alcohol exposure. Imaging and neurobehavioral research in individuals with FASD have revealed that some brain regions are particularly sensitive to prenatal alcohol exposure, whereas other areas apparently are relatively more spared. Particularly vulnerable regions include the frontal cortex, caudate, hippocampal formation, corpus callosum, and components of the cerebellum, including the anterior cerebellar vermis. One technology that is relatively new for analyzing the effects of prenatal alcohol exposure is a specific form of MRI called diffusion tensor imaging (dtMRI). This technique allows investigators to visually track changes in bundles of nerve fibers (i.e., white matter tracts) in the brains of humans at any age. These tracts now are known to be adversely affected by prenatal alcohol exposure and may relate to alterations in information processing. In addition, another MRI technology called functional MRI (fMRI) is being used to link behavioral and cognitive deficits with alterations in the functioning of certain CNS regions.

Other imaging techniques, such as magnetic resonance spectroscopy (MRS), positron emission tomography (PET), and single-photon emission computed tomography (SPECT) indicate that prenatal alcohol exposure alters metabolite levels and ratios, cerebral blood flow, and brain signaling (i.e., neurotransmitter) systems—changes that may be evident even in the absence of gross morphological changes. Moreover, brain imaging may be useful for detecting neuropathology early in development, and prenatal and/or neonatal ultrasound analyses may be used to identify developmental disruptions, thereby helping to improve early diagnosis and intervention efforts. Finally, functional neuroimaging studies also may become useful in contributing to the diagnosis of children with FASD as well as monitoring the efficacy of various interventions (for more information, see the article by Nuñez and colleagues, pp. 121–131 in this issue).

Researchers also are using brain-imaging techniques in animal studies of FASD. Three-dimensional brain reconstruction using imaging technologies provides a powerful tool to complement standard techniques looking at stained tissue slices (i.e., histological techniques). In animal models, these strategies allow investigators to directly manipulate alcohol exposure parameters and measure the resultant neuropathology. For example, the extent of damage to any brain area may be related to the timing of alcohol exposure relative to developmental processes (i.e., neurogenesis) that are occurring in that particular brain region, as well as to the overall stage of embryonic development (Guerri et al. 2009). Manipulation of such variables is helping researchers to better account for variability observed in outcomes of individuals with FASD. Moreover, measures that are similar to those obtained in clinical studies now also can be obtained in animal models, facilitating translation of findings among species. (see O’Leary-Moore et al., pp. 99–105, in this issue).

### Mechanisms of Alcohol’s Prenatal Effects

Alcohol seems to have many distinct actions through which it can induce harm to the developing embryo and fetus and which, depending on the developmental timing of exposure, contribute to the variability of outcomes observed across the spectrum of prenatal alcohol effects. Animal models are providing important insights into how alcohol causes both structural and functional damage. Evidence for alcohol-induced alterations has been found throughout pregnancy, even as early as the embryonic stage of development. For example, using an embryonic frog model representing very early gestation, researchers demonstrated that alcohol can induce developmental injury by decreasing the expression of several key genes necessary for development (Yelin et al. 2007). This reduced gene expression results in outcomes consistent with those seen in FAS, including smaller head size (i.e., microcephaly), abnormally small eyes (i.e., microphthalmia) and other ocular abnormalities, overall growth retardation, and delayed gut development. Furthermore, Yelin and colleagues (2007) demonstrated that highly reactive oxygen-containing molecules (i.e., reactive oxygen species) and potentially reactive nitrogen species are involved in the mechanisms by which alcohol causes these developmental effects. Consistent with these observations, the antioxidant ascorbic acid (vitamin C) can protect against alcohol-related microcephaly and overall growth impairment (Peng et al. 2005).

Programmed cell death (i.e., apoptosis) is an essential process for normal development. If, however, apoptotic events occur too early, too late, or in excess, the developmental trajectory can be altered. Both in vivo and in vitro studies have demonstrated that oxidative stress induced by alcohol may cause apoptosis, in part by reducing the cells’ levels and activity of antioxidants. These findings have led investigators to test whether antioxidants could prevent fetal alcohol injury. The results of such studies to date have been mixed but demonstrate that some antioxidants can partially protect against alcohol-induced developmental damage (Da Lee et al. 2005).

Another important functional system altered by prenatal alcohol exposure is the L1 cell adhesion system. Like other adhesion molecules, the L1 cell adhesion molecule (L1CAM) helps cells bind with each other or with other large molecules outside the cell, thereby guiding the growth of cells and ensuring the formation of functional tissues. It is interesting to note that children who are born with mutations involving the L1 molecule develop birth defects similar to those seen in children with FAS (Yeaney et al. 2009). Studies found that even low alcohol concentrations, such as those resulting after consumption of one drink, can interfere with the ability of L1CAM to mediate cell adhesion and axonal growth (Ramanathan et al. 1996). These findings illustrate that alcohol can interfere with communication among cells and molecules during development and indicate additional targets for intervention to prevent or ameliorate alcohol-related birth defects.

In addition to alcohol directly disrupting development, there also is evidence that alcohol withdrawal may be damaging to the developing fetus. This pathway of damage may involve the brain-signaling molecule (i.e., neurotransmitter) glutamate and the molecules (i.e., receptors) with which it interacts, specifically a receptor called the *N*-methyl-d-aspartate (NMDA) receptor. Alcohol may acutely block NMDA receptors, to which the body responds by increasing the activity of those receptors in order to compensate for alcohol’s effects. When the alcohol is withdrawn, however, this compensation results in an overactivation of NMDA receptors. In fact, administration of various agents that prevent NMDA receptor activation (i.e., NMDA receptor antagonists), including MK-801, eliprodil, agmatine, and memantine, during the withdrawal period can protect against neuropathology and behavioral deficits associated with developmental alcohol exposure (Lewis et al. 2007; Thomas et al. 2001, 2004).

Finally, prenatal alcohol exposure may exert its harmful effects via epigenetic mechanisms. These are mechanisms that alter the activity (i.e., expression) of certain genes without, however, changing the genes’ DNA sequence. Examples of epigenetic mechanisms include methylation of the DNA and/or changes to the way the DNA is packaged in the cell’s nucleus in the form of DNA–protein complexes (i.e., chromatin). Alcohol-induced epigenetic changes during the prenatal period might account for some of the deleterious effects resulting from prenatal alcohol exposure (for more information, see the article by Kobor and Weinberg, pp. 29–37 in this issue.)

In summary, it is clear that alcohol disrupts developmental processes through multiple sites of action. Establishing the mechanisms through which alcohol causes FASD and applying this knowledge to the prevention and treatment of these disorders is an ongoing challenge for scientists.

### Prevention and Intervention

Despite health warnings issued by the Federal Government since 1977 (Food and Drug Administration 1977, 1981; U.S. Surgeon General 2005), including point-of-sale warning signs and bottle labeling, women who are pregnant and women of childbearing age continue to drink and to drink in patterns that significantly increase risk for FASD among their offspring. Among women who are in their childbearing years who might become pregnant, 55 percent reported using alcohol in the previous month, 12.4 percent reported consuming five or more drinks on one occasion (i.e., binge drinking), and 13.1 percent reported either drinking seven or more drinks in a week or binge drinking (i.e., frequent use) (Rasmussen et al. 2009). This particularly is important for nonpregnant women of childbearing age because one-half of pregnancies in the United States are unplanned (Finer and Henshaw 2006). In these cases, a woman may be unaware that she is pregnant and therefore may continue to drink during the early stages of pregnancy, placing her fetus at risk for FASD. In a recent analysis of data from the Behavioral Risk Factor Surveillance System surveys, the CDC (2009) found that the prevalence of previous month alcohol use and binge drinking among pregnant and nonpregnant women of child-bearing age did not change substantially from 1991 to 2005. In another study, when women were asked about drinking at any time during pregnancy (rather than during the past 30 days), 30.3 percent of women reported drinking at some time during pregnancy and 5.7 percent reported binge drinking (Rasmussen et al. 2009). There clearly is a need to bridge the gap between knowledge and behavior. Understanding why some women continue to drink even when they know that it may be potentially harmful remains a key challenge, as is the identification of effective interventions to reduce the risk of a child developing FASD.

Prevention methods may be improved by identifying factors that convey protection from or increase risk of FASD. Identification of such factors not only may help elucidate the mechanisms of alcohol-induced birth defects but may help target prevention and intervention strategies to populations at highest risk. For example, genetic factors can influence the extent of adverse pregnancy outcome in both humans and animal models. Animal research has shown that the genetic profiles of the mother and the fetus are important for determining the potential for risk of alcohol-related physical birth defects, prenatal mortality, and learning and other neurobehavioral deficits in the offspring (for more information, see the article in this issue by Wilson and Cudd, pp. 92–98). In humans, the presence of a specific variant of the gene encoding the alcohol-metabolizing enzyme alcohol dehydrogenase (ADH)1, called ADH1B*2, in either the mother or child decreases the risk for FAS (see Warren and Li 2005). Other studies have shown that the presence of another ADH1 variant, ADH1B*3, decreases the risk for neurodevelopmental deficits associated with FASD (see Warren and Li 2005). Both of these ADH variants are more efficient at oxidizing alcohol to acetaldehyde, a toxic intermediate metabolite, suggesting that elevated acetaldehyde levels may contribute to decreased alcohol consumption, which, in turn, lessens the risk of FASD (Guerri et al. 2009; Warren and Li 2005; for more information, see the article by May and Gossage, pp. 15–26 in this issue).

In addition to searching for factors that influence risk of FASD and which might be targets for prevention efforts, researchers are pursuing two other paths for preventing FASD—elimination or reduction of alcohol consumption by pregnant women and interventions for alcohol-exposed individuals to prevent or reduce alcohol’s harmful effects on the fetus.

#### Preventing Potentially Harmful Alcohol Consumption

The most desirable prevention approach involves eliminating or significantly reducing alcohol consumption by women during pregnancy. The IOM has proposed several strategies targeting different population subgroups that continue to form the basis of prevention strategies aimed at reducing the prevalence of FASD (Stratton et al. 1996). These can be classified as universal, selected, and targeted strategies. Universal approaches are aimed at all members of a population (e.g., all women) and typically involve general information and education. Examples of such universal approaches include notices in bars, restaurants, and other points of sale; broad media campaigns; and warning labels on alcohol beverage containers. Research to date has not demonstrated that universal approaches decrease alcohol use among the group at highest risk for having a child with FASD.

Selected approaches are directed to women who are in special risk groups (e.g., women who frequently engage in binge drinking). An example of such selective approaches would be screening efforts in primary care or prenatal clinics in communities known to have a high prevalence of risky drinking. Indicated or targeted prevention is directed at women known to be more vulnerable because they frequently drink in a high-risk manner (e.g., frequent binge drinking), have received a diagnosis of alcoholism, or have previously given birth to a child with FASD.

Limited research on selected and targeted prevention efforts has shown that these methods may produce changes in drinking behavior that potentially can decrease the risk for an adverse fetal outcome. For instance, studies found that both screening for alcohol use and administration of brief interventions in the clinic (both of which are considered selected prevention approaches) have positive effects on drinking reduction during pregnancy (Chang 2004; Chang et al. 2005). Several screening instruments offer good sensitivity and specificity in identifying women at risk, and their effectiveness may be enhanced by computerized self-interview (Dawson et al. 2001).

#### Interventions for Alcohol-Exposed Individuals

Other efforts are exploring the possibility of minimizing the damage caused by prenatal alcohol exposure. Pharmacological intervention during pregnancy is one prevention approach that may be particularly suitable if a woman consumed alcohol before she realized she was pregnant or if she otherwise fails to stop drinking in pregnancy. Unlike many other teratogens that have limited periods of exposure vulnerability, alcohol can produce embryonic and fetal injury during multiple gestational periods. Promising agents that have been shown to reduce fetal cell toxicity include anti-inflammatory agents such as prostaglandin inhibitors, growth factors, antioxidants, the nutrient choline, and agents that interfere with alcohol-related disruption of the L1CAM system (Yeaney et al. 2009). Also, two small molecules (i.e., neuropeptides) derived from neurotrophic factors produced by the body that induce survival, development, and function of nerve cells (i.e., activity-dependent neurotrophic factor and activity-dependent neuroprotective protein) have been shown to provide significant protection from alcohol-induced fetal injury in cell culture and animal models (Sari and Gozes 2006). Thus, derivatives of these neurotrophic factors may offer significant potential as future protective agents.

It should be noted that although the field now is identifying a number of potential effective treatments, challenges exist. First, although agents may ameliorate alcohol’s effects on a specific targeted system in a given study, it is not likely that any single intervention will address alcohol’s many teratogenic mechanisms of action. Second, any pharmacotherapeutic intervention itself may have the potential to induce additional teratogenic effects if nontargeted developmental events are affected.

To develop more effective interventions, researchers must enhance understanding of the mechanisms by which alcohol causes damage, of the factors that ameliorate or exacerbate these effects (e.g., critical periods for alcohol exposure or maternal nutritional status), and how these factors relate to levels of alcohol exposure and consequences.

### Therapeutics

Several promising approaches to restore or improve neurobehavioral outcome in individuals who have been exposed to alcohol prenatally are being explored both clinically and in animal models (for more information, see the article by Paley and O’Connor, pp. 64–75, and Idrus and Thomas, pp. 76–85 in this issue.) For example, complex motor skills training in adult rats can mitigate performance deficits on a task that resulted from binge exposure to alcohol during development (Klinstova et al. 2000). The analyses also demonstrated that this training stimulates the formation of new connections among nerve cells (i.e., synaptogenesis) in the cerebellum. Other forms of directed activity may have similar beneficial effects in other neuronal cell populations.

Nutritional interventions also may be effective later in life. Dietary supplementation with the nutrient choline can decrease hyperactivity and improve spatial and working memory in rats that had been exposed to alcohol during development (Thomas et al. 2007). Postnatal choline administration may be effective, in part, by acting as a precursor to the neurotransmitter acetylcholine. Alcohol-induced changes in cholinergic functioning in a brain region called the hippocampus can lead to hyperactivity, passive avoidance learning deficits, and impairments in spatial and working memory. Of interest, postnatal choline treatment had no effect on alcohol-induced deficits in motor balance and coordination, suggesting that choline’s ameliorative effects when administered after alcohol exposure may be more effective for hippocampal dysfunction.

One of the main characteristics of the brain is the ability to adapt to changes throughout life. This process, which is called neuronal plasticity, is the basis for life-long learning. Elucidation of how prenatal alcohol exposure influences life-long neuronal plasticity is critical for identifying pharmacological, nutritional, educational, or behavioral interventions that can capitalize on that plasticity. The hope is that if prevention strategies fail to eliminate FASD, improved and targeted interventions can improve the quality of life of individuals with FASD.

## Summary

Alcohol research has made great strides toward understanding the causes and consequences of prenatal exposure to alcohol since its initial clinical description over three decades ago. The present and future challenges will be to further refine both basic and clinical research to improve case recognition for FASD, both through the identification of biomarkers and through a better understanding of the neurodevelopmental characteristics that define FASD, and to apply this knowledge of neurobehavioral phenotype to the development of appropriate clinical and educational interventions. Other efforts need to center on enhancing prevention through increased screening for alcohol use, better education of health care professionals, and societal changes that will help to influence the behavior of women who are pregnant and who are in their childbearing years. Finally, it will be crucial to improve understanding of the genetic, socioeconomic, age, and other factors involved in the development of FASD to better target preventive and treatment approaches where they will be most effective.

## Figures and Tables

**Figure:**
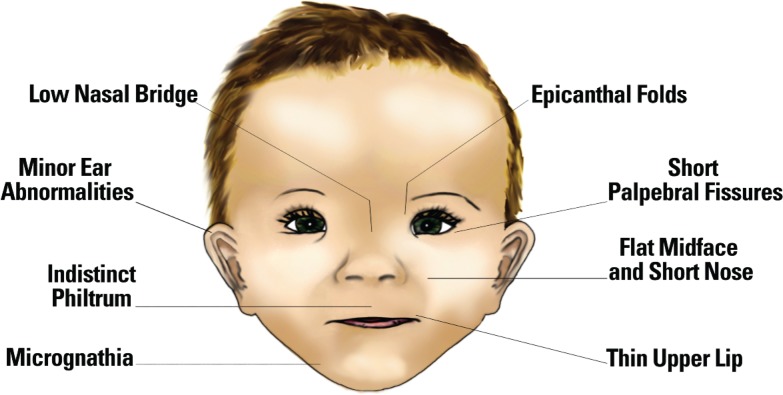
Facial characteristics that are associated with fetal alcohol exposure.

**Table:** Summary and Comparison of the Various Diagnostic Schemas for Prenatal Alcohol Related Disorders

	**4-Digit Code^5^**	**Revised IOM^6^**	**Canadian^7^**	**National Task Force on FAS/FAE^8^**
**FAS**				
Facial Characteristics	Simultaneous presentation of short palpebral fissures (≤ 2 SDs), thin vermillion border, smooth philtrum.	Two of the following: short palpebral fissures (≤10th percentile), thin vermillion border, smooth philtrum.	Simultaneous presentation of short palpebral fissures (≤ 2 SDs), thin vermillion border, smooth philtrum.	Simultaneous presentation of short palpebral fissures (≤10th percentile), thin vermillion border, smooth philtrum.
Growth Retardation	Height or weight ≤10th percentile.	Height or weight ≤10th percentile.	Height or weight or disproportionately low weight-to-height ratio (≤10th percentile).	Height or weight ≤ 10th percentile.
Central nervous system (CNS) involvement	Head circumference (occipital-frontal circumference [OFC]) ≥ 2 SDs below norm or significant abnormalities in brain structure or evidence of hard neurological findings or significant impairment in three or more domains of brain function (≥2 SDs below the mean) as assessed by validated and standardized tools.	Head circumference (OFC) ≤10th percentile or structural brain abnormality.	Evidence of three or more impairments in the following CNS domains: hard and soft neurologic signs; brain structure; cognition; communication; academic achievement; memory; executive functioning and abstract reasoning; attention deficit/hyperactivity; adaptive behavior, social skills, social communication.	Head circumference (OFC) ≤10th percentile or structural brain abnormality or neurological problems or other soft neurological signs outside normal limits or functional impairment as evidenced by global cognitive or intellectual deficits, below the 3rd percentile (2 SDs) below the mean or functional deficits below the 16th percentile (1 SD) below the mean in at least three domains: cognitive or developmental markers, executive functioning, motor, social skills, attention/hyperactivity, and other (i.e. sensory, memory, language).
Alcohol Exposure	Confirmed or not confirmed.	Confirmed or not confirmed.	Confirmed or not confirmed.	Confirmed or not confirmed.
**Partial FAS**				Not proposed
Facial Characteristics	Short palpebral fissures (≤2 SDs) and either a smooth philtrum or thin vermillion border, with the other being normal OR palpebral fissure (≤1 SD) and both a smooth philtrum and thin vermillion.	Two or more of the following: short palpebral fissures (≤10th percentile), thin vermillion border, smooth philtrum.	Two or more of the following: short palpebral fissures, thin vermillion border, smooth philtrum.	Not applicable
Growth Retardation	Not required	Either height or weight ≤10th percentile OR (see CNS involvement).	Not required	Not applicable
Central nervous system (CNS) involvement	Same as for FAS	Head circumference ≤10th percentile or structural brain abnormality or behavioral and cognitive abnormalities inconsistent with developmental level.	Same as for FAS	Not applicable
Alcohol Exposure	Confirmed	Confirmed or not confirmed	Confirmed	Felt that there was insufficient data to provide guidance for this diagnosis. Formed group to discuss.
**ARND**	Does not propose this diagnostic category, but rather has several categories assessing functional deficits.			Not applicable
Central nervous system involvement	Same as for FAS	Either 1) structural brain anomaly or OFC ≤10th percentile or 2) evidence of a complex pattern of behavioral or cognitive abnormalities inconsistent with developmental level that cannot be explained by genetics, family background or environment alone.	Same as for FAS	Not applicable
Alcohol Exposure	Confirmed	Confirmed	Confirmed	Not applicable
Notes	The 4-Digit Code provides an assessment of effects in four areas (growth, face, CNS, and alcohol exposure) that results in 256 different codes and 22 diagnostic categories. A specific pattern or level of alcohol exposure is not required, just that alcohol exposure is confirmed or not.	Alcohol exposure is defined as a pattern of excessive intake or heavy episodic drinking.	Alcohol exposure is defined as a pattern of excessive intake or heavy episodic drinking. A domain is considered “impaired” when on a standardized measure: Scores are ≥ 2 SDs below the mean, or there is a discrepancy of at least 1 SD between subdomains or there is a discrepancy of at least 1.5–2 SD among subtests on a measure.	Alcohol exposure levels are not defined, but the authors cite evidence of alcohol exposure based upon clinical observation; self-report; reports of heavy alcohol use during pregnancy by a reliable informant; medical records documenting positive blood alcohol levels, or alcohol treatment; or other social, legal, or medical problems related to drinking during pregnancy.

1. All of the diagnostic schemes assume that genetic or medical causes have been ruled out and that appropriate norms are used when available.

2. All of the diagnostic schemes use the University of Washington Lip-Philtrum Guide (http://depts.washington.edu/fasdpn/htmls/lip-philtrum-guides.htm).

3. For palpebral fissure norms, the 4-Digit Code uses Hall et al. 1989, Hoyme utilizes Thomas et al. 1987, and Chudley provides both the Thomas and Hall charts; the National Task Force guidelines do not mention which chart to use. Hall recently wrote that her charts underrepresented normal palpebral fissure length (Hall 2010) and should be replaced by those from Clarren et al. 2010.

4. Note that < 2 SD = 2.3rd percentile in a normal distribution

5. Astley and Clarren 2000

6. Hoyme et al. 2005

7. Chudley et al. 2005

8. Bertrand et al. 2004

SOURCE: Modified with permission from Riley et al. 2011.

## Footnotes

1 According to this classification, a person can manifest both ARBD and ARND, without, however, meeting the criteria for full FAS.

2 The sensitivity of a diagnostic tool is a measure of how often it correctly identifies patients with a given condition (i.e., the proportion of people with the condition who are correctly identified as such); conversely, the specificity is a measure of the tool’s ability to correctly identify patients who do not have the condition of interest (i.e., individuals with a developmental disorder that is not FASD).
